# Strategic Priorities to Increase Use of Clinical Preventive Services Among Older US Adults

**DOI:** 10.5888/pcd10.120231

**Published:** 2013-04-11

**Authors:** Amy Slonim, William Benson, Lynda A. Anderson, Ellen Jones

**Affiliations:** Author Affiliations: William Benson, Health Benefits ABCs (Consultant to CDC’s Healthy Aging Program), Atlanta, Georgia; Lynda A. Anderson, Centers for Disease Control and Prevention, Atlanta, Georgia; Ellen Jones, University of Mississippi Medical Center, Jackson, Mississippi.

## Abstract

The objective of this project was to obtain professionals’ perceptions of system-level strategies with potential to increase use of clinical preventive services (CPS) among adults aged 50 years or older through community settings. Public health, aging services, and medical professionals participated in guided discussions and a modified Delphi process. Priority strategies, determined on the basis of a 70% or higher a priori agreement level, included enhancing community capacity; promoting the design of health information technologies to exchange data between clinical and community settings; promoting care coordination; broadening scope of practice; providing incentives to employers; and eliminating cost-sharing. Findings provide insights about preferences for system-level strategies that align with national and state initiatives to increase CPS use.

## Objective

Prevention initiatives highlight the need to increase clinical preventive services (CPS) use among adults through community settings (1–7). Less than 50% of adults aged 50 years or older are current on select recommended screenings and vaccinations (2,5). Increasing the use of recommended CPS can help with detecting chronic conditions, delaying their onset, or identifying them early in their most treatable states (8). Innovative system-level strategies are needed to promote CPS through community settings linked to clinical efforts (5). Our objective was to determine professionals’ perceptions about system-level strategies with potential to increase CPS use among adults aged 50 years or older in or through community settings.

## Methods

We used qualitative methods, including a key informant approach, to elicit input from professionals throughout the country. We sought balanced representation among 1) people with national, state, and local perspectives; 2) workers from public and private sectors; and 3) public health, aging services, and medical professionals. Professionals were told their responses would be confidential and used in the development of a public health practice project for the National Association of Chronic Disease Directors.

### Phase 1 (May–August 2010)

Authors (A.S., W.B.) led guided one-on-one telephone discussions with 25 professionals to elicit strategies designed to expand on opportunities to provide or overcome barriers that limit CPS delivery (Figure). We used thematic analysis to identify a set of overarching strategies, which were categorized into discrete topics, reviewed to eliminate duplication, and refined to be mutually exclusive.

**Figure:**
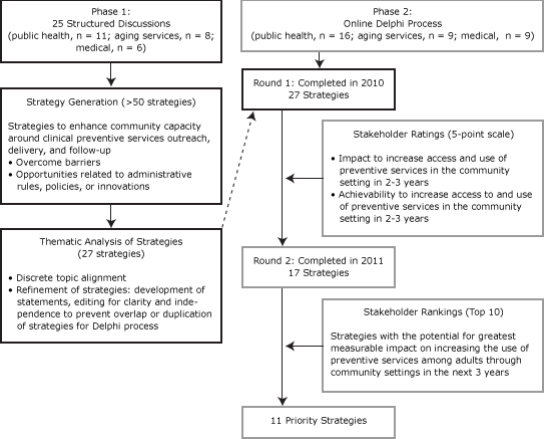
Methods and participation rates for Phase 1 (May to August 2010) and 2 rounds of an online Delphi process (9), Phase 2 (November 2010 to January 2011), to derive 11 priority strategies to increase the use of clinical preventive services.

### Phase 2 (November 2010–May 2011)

We invited 45 professionals, most of whom participated in Phase 1, to participate in a modified Delphi process using SurveyMonkey (SurveyMonkey, Palo Alto, California). Delphi is a structured method to facilitate agreement among experts who have diverse perspectives (9). In round 1, panelists received background information and a list of 27 strategies that were generated during Phase 1 discussions for increasing the use of CPS, ordered randomly to reduce potential bias. Strategies were rated on a 5-point scale reflecting potential impact and achievability (1 being the lowest to 5 being the highest). Three scores were calculated: 1) a mean score for potential impact, 2) a mean score for achievability, and 3) the mean of a summed score that combined the individual impact and achievability scores. For round 2, we decided in advance to retain strategies with average scores of 3.5 or higher on achievability alone or on the joint rating. In round 2, we provided average ratings for each strategy from round 1. Panelists responding to round 1 ranked the 10 strategies they considered to have the greatest measurable impact on increasing use of CPS in the next 3 years (Figure). Priorities were defined as those strategies ranked in the top 10 by 70% or more of respondents.

## Results

Of 42 professionals, 34 (81%) completed Phase 2. The final panel members included 18 national, 5 state, and 11 local representatives; 56% were from the private sector. Sixteen professionals were from public health, 9 were from aging services, and 9 were medical professionals (15 men and 19 women).

During round 1, mean panelist ratings for achievability for the 27 strategies ranged from 2.6 to 3.9, and mean ratings for impact ranged from 2.6 to 4.2. The joint average score combining impact and achievability ratings ranged from 2.6 to 4.0. A total of 15 strategies received scores of 3.5 or higher on the joint average score combining impact and achievability, and 2 strategies received scores of 3.5 or higher for achievability alone. Therefore, 17 of the 27 strategies were included in the second round. (The list of 17 strategies is available on request from the corresponding author.)

In round 2, all 17 strategies were rated in the top 10 by at least 1 panelist. Three strategies were rated in the top 10 by all panelists (column 4, Table). These strategies were 1) expand eligibility for public and private funding for CPS in nontraditional settings; 2) for those services recommended by the US Preventive Services Task Force (8), eliminate cost-sharing covered by the public sector; and 3) promote inclusion of CPS in care coordination models.

**Table:** Respondents’ Priority Strategies to Increase Clinical Preventive Services Among US Adults Through Community Settings, December 2010–January 2011^a^

Priority Strategies	Percentage of Respondents That Ranked Each Strategy Among Their Top 10 Priorities			
	Public Health Professionals (n = 16)	Aging Services Professionals (n = 9)	Medical Professionals (n = 9)	Total (n = 34)
Expand eligibility for public and private funding for preventive services delivery to include nonclinical, community-based organizations and nontraditional settings.	75		78	74
Eliminate cost-sharing for all preventive services that receive an A or B rating by the United States Preventive Services Task Force covered by the public sector.	81			71
Promote inclusion of preventive services in care coordination models.	75			71
Amend scope of practice laws to allow appropriate allied health professionals to provide specified screenings and preventive services counseling of older adults.	100			
Eliminate cost-sharing for all preventive services that receive an A or B rating by the United States Preventive Services Task Force covered by the private insurance providers and plans.	81			
Strengthen the capacity of the aging services network (established by the Older Americans Act) to work collaboratively with the public health system to promote and coordinate the delivery of preventive services in community settings conducting demonstration programs under Title IV (10).		100		
Strengthen the capacity of the aging services network to work collaboratively with the public health system to promote and coordinate the delivery of preventive services in community settings by including such activities in the Health Promotion and Disease Prevention section of the Older Americans Act (Title III, Part D) (10).		78		
Strengthen the promotion of positive health behaviors and outcomes, including the use of preventive services, in medically underserved communities through the Community Health Workforce competitive grants program (Patient Protection and Affordable Care Act, Section 5313 [11]).		78		
Promote incentives for employers to provide preventive services on-site.			89	
Increase the availability of programs designed to expand access to community-based preventive services through the Prevention and Public Health Fund (Patient Protection and Affordable Care Act, Section 4002 [11]).			89	
Design electronic medical records and health information technology system that ensure patient information related to preventive services is exchanged securely and reliably between clinical and community settings.			78	

a Participants ranked priority areas for their potential to have the greatest measurable impact on increasing use of clinical preventive services; empty cells indicate that the strategy was not ranked ≥70% by respondents in that group.

Examination of the findings in 3 professional subgroups (public health, aging services, and medical professionals) indicated that 11 strategies (65%) were among the top 10 identified by 1 or more of the subgroups (columns 1-3, Table). However, no single strategy met the 70% or higher criteria across all 3 subgroups; only 1 strategy was chosen by more than 1 of the 3 professional subgroups as a priority strategy.

## Discussion

We found that members of diverse professional groups chose priority strategies to address the following areas: strengthening community capacity to deliver CPS, expanding or promoting the design of health information technologies to exchange data between clinical and community settings, eliminating cost-sharing, promoting inclusion in care coordination, addressing scope of practice to broaden allied health professionals’ roles in providing CPS, and promoting incentives to employers to provide CPS. Although no single strategy ranked highly across the 3 professional subgroups, all prioritized strategies link to key national initiatives including the National Prevention Strategy (1), the Older Americans Act (10), the Patient Protection and Affordable Care Act (11), and a Purchaser’s Guide to Clinical Preventive Services (12), or they link to state initiatives (13).

The project and associated modified Delphi process has several advantages and limitations (9,14). Advantages include respondent anonymity, Web-based processes, and resulting rapid understanding gained from diverse expertise and geographic locations. Limitations include lack of generalizability and limited understanding of rationale for the selected priorities. Furthermore, we did not continue the process until there was complete consensus. Representative groups, such as payers, did not participate, so their perspectives are not represented.

This is the first documented work of which we are aware to elicit perspectives among these professional groups about system-level strategies believed to be helpful in increasing use of CPS among older adults. These strategies, which align with national and state initiatives, provide insights into stakeholders’ preferences for specific sets of system-level strategies. Creating a conceptual framework on the basis of these diverse perspectives that cohesively integrates community and clinical efforts to increase use of CPS would be valuable.

## References

[1] National Prevention Council. National prevention strategy. Washington (DC): US Department of Health and Human Services, Office of the Surgeon General; 2011. http://www.surgeongeneral.gov/initiatives/prevention/strategy/report.pdf. Accessed July 2012.

[2] Healthy people 2020: older adult objectives. US Department of Health and Human Services, Office of Disease Prevention and Health Promotion. http://healthypeople.gov/2020/topicsobjectives2020/objectiveslist.aspx?topicid=31. Accessed July 25, 2012.

[3] Centers for Disease Control and Prevention. Guide to community preventive services. The Task Force on Community Preventive Services. http://www.thecommunityguide.org. Accessed July 25, 2012.

[4] Million hearts. US Department of Health and Human Services, Centers for Disease Control and Prevention, Centers for Medicaid and Medicare Services. http://millionhearts.hhs.gov/index.html. Accessed July 25, 2012.

[5] Promoting preventive services for adults 50–64: community and clinical partnerships. Centers for Disease Control and Prevention; 2009. http://www.cdc.gov/aging/pdf/promoting-preventive-services.pdf. Accessed July 25, 2012.

[6] Enhancing use of clinical preventive services among older adults. Centers for Disease Control and Prevention; 2011. http://www.cdc.gov/features/preventiveservices/clinical_preventive_services_closing_the_gap_report.pdf. Accessed July 25, 2012.

[7] Community transformation grants. http://www.cdc.gov/communitytransformation/. Centers for Disease Control and Prevention, Division of Community Health. Accessed July 25, 2012.

[8] Recommendations. US Preventive Services Task Force. http://www.uspreventiveservicestaskforce.org/recommendations.htm. Accessed February 20, 2013.

[9] Clayton MJ . Delphi: a technique to harness expert opinion for critical decision-making tasks in education. Educ Psychol 1997;17(4):373–86. 10.1080/0144341970170401

[10] Older Americans Act, 42 USC Chapter 3. http://uscode.house.gov/download/pls/42C35.txt. Accessed July 25, 2012.

[11] Patient Protection and Affordable Care Act of 2010, Pub L No 111-148, 124 §3021, 4002, 4201, 4103, 5405, 5601, 9007 (2010).

[12] Campbell KP , Lanza A , Dixon R , Chattopadhyay S , Molinari N , Finch RA , editors. A purchaser’s guide to clinical preventive services: moving science into coverage. Washington (DC): National Business Group on Health; 2006.

[13] LeBuhn R , Swankin DA . Reforming scopes of practice: a white paper. Washington (DC): Citizen Advocacy Center; 2010.

[14] Rao JK , Alongi J , Anderson LA , Jenkins L , Stokes GA , Kane M . Development of public health priorities for end-of-life initiatives. Am J Prev Med 2005;29(5):453–60. 10.1016/j.amepre.2005.08.014 16376710

